# *KIBRA* Gene Variant Is Associated with Ability in Chess and Science

**DOI:** 10.3390/genes14010204

**Published:** 2023-01-13

**Authors:** Ildus I. Ahmetov, Elena V. Valeeva, Meruert B. Yerdenova, Gaukhar K. Datkhabayeva, Amal Bouzid, Poorna Manasa Bhamidimarri, Liliya M. Sharafetdinova, Emiliya S. Egorova, Ekaterina A. Semenova, Leysan J. Gabdrakhmanova, Rinat A. Yusupov, Andrey K. Larin, Nikolay A. Kulemin, Edward V. Generozov, Rifat Hamoudi, Almira M. Kustubayeva, Tim Rees

**Affiliations:** 1 Research Institute for Sport and Exercise Sciences, Liverpool John Moores University, Liverpool L3 5AF, UK; 2 Laboratory of Genetics of Aging and Longevity, Kazan State Medical University, 420012 Kazan, Russia; 3 Department of Molecular Biology and Genetics, Federal Research and Clinical Center of Physical-Chemical Medicine of Federal Medical Biological Agency, 119435 Moscow, Russia; 4 Department of Physical Education, Plekhanov Russian University of Economics, 115093 Moscow, Russia; 5 Department of Psychology, Al-Farabi Kazakh National University, Almaty 050040, Kazakhstan; 6 Department of Biophysics, Biomedicine and Neuroscience, Center for Cognitive Neuroscience, Al-Farabi Kazakh National University, Almaty 050040, Kazakhstan; 7 Sharjah Institute for Medical Research, College of Medicine, University of Sharjah, Sharjah 27272, United Arab Emirates; 8 Research Institute of Physical Culture and Sport, Volga Region State University of Physical Culture, Sport and Tourism, 420138 Kazan, Russia; 9 Department of Physical Culture and Sport, Kazan National Research Technical University Named after A.N. Tupolev-KAI, 420111 Kazan, Russia; 10 Division of Surgery and Interventional Science, University College London, London NW3 2PF, UK; 11 Department of Rehabilitation and Sport Science, Faculty of Health and Social Sciences, Bournemouth University, Bournemouth BH12 5BB, UK

**Keywords:** DNA, memory, education, PhD, intelligence, chess, mind sports, analytical ability, scientists, athletes

## Abstract

The kidney and brain expressed protein (KIBRA) plays an important role in synaptic plasticity. Carriers of the T allele of the *KIBRA* (*WWC1*) gene rs17070145 C/T polymorphism have been reported to have enhanced spatial ability and to outperform individuals with the CC genotype in working memory tasks. Since ability in chess and science is directly related to spatial ability and working memory, we hypothesized that the *KIBRA* T allele would be positively associated with chess player status and PhD status in science. We tested this hypothesis in a study involving 2479 individuals (194 chess players, 119 PhD degree holders in STEM fields, and 2166 controls; 1417 males and 1062 females) from three ethnicities (236 Kazakhs, 1583 Russians, 660 Tatars). We found that frequencies of the T allele were significantly higher in Kazakh (66.9 vs. 55.1%; *p* = 0.024), Russian (44.8 vs. 32.0%; *p* = 0.0027), and Tatar (51.5 vs. 41.8%; *p* = 0.035) chess players compared with ethnically matched controls (meta-analysis for CT/TT vs. CC: OR = 2.05, *p* = 0.0001). In addition, none of the international chess grandmasters (ranked among the 80 best chess players in the world) were carriers of the CC genotype (0 vs. 46.3%; OR = 16.4, *p* = 0.005). Furthermore, Russian and Tatar PhD holders had a significantly higher frequency of CT/TT genotypes compared with controls (meta-analysis: OR = 1.71, *p* = 0.009). Overall, this is the first study to provide comprehensive evidence that the rs17070145 C/T polymorphism of the *KIBRA* gene may be associated with ability in chess and science, with the T allele exerting a beneficial effect.

## 1. Introduction

The kidney and brain expressed protein (KIBRA), together with its binding partners (dendrin, synaptopodin, dynein-complex and others), plays an important role in synaptic plasticity [1], which enables the brain to store and recall information. KIBRA is considered a postsynaptic scaffold protein connecting cytoskeletal and signaling molecules, with high expression in memory- and spatial learning-related regions of the brain (cortex, hippocampus), as well as in the cerebellum and hypothalamus [2].

KIBRA attracted the attention of behavioral geneticists following the publication of a 2006 genome-wide association study (GWAS) of memory performance and executive function [3]. In that study, the T allele of the *KIBRA* (also known as *WWC1*) gene rs17070145 C/T polymorphism was positively associated with episodic memory performance in three independent cohorts [3]. Subsequent studies have examined the rs17070145 single nucleotide polymorphism (SNP) with respect to cognitive performance in different populations [1,4]. Here, *KIBRA* T allele carriers have been shown to outperform individuals with the CC genotype in episodic and working memory tasks [4,5,6]. Furthermore, the T allele has been linked with increased cognitive performance [7] and better scores in spatial ability [8], spatial learning [9], and navigational success [10].

Spatial ability is a highly heritable (84%) trait [11] and is a predictor of success in science, technology, engineering, and mathematics (i.e., STEM subjects) and other mentally demanding fields [12,13]. Studies suggest that a high level of spatial ability, working-memory capacity, and other cognitive abilities are necessary to reach a high standard of performance in chess [14,15,16]. Working memory is also a heritable (31–72%) trait [17,18] and is positively associated with analytic problem-solving [19] and fluid intelligence [20], suggesting that working memory capacity may be a contributor to achievement in a scientific career.

Because ability in chess and science both appear to be related to spatial ability and working memory, we hypothesized that the *KIBRA* rs17070145 T allele might be positively associated with chess player status and PhD status in science. Thus, in the present and first study of its type, our aim was to compare allelic frequencies of the *KIBRA* rs17070145 C/T polymorphism between chess players, PhD holders, and controls. Through this novel focus, and by comprehensively examining these comparisons in three independent cohorts, combined with follow-up meta-analyses of our results, we sought to make a unique contribution to the field.

## 2. Materials and Methods

### 2.1. Ethics Statement

The Ethics Committee of the Federal Research and Clinical Center of Physical-Chemical Medicine of the Federal Medical and Biological Agency of Russia (Approval number 2017/04) and the Ethics Committee of the Al-Farabi Kazakh National University (Approval numbers: IRB-A172 and IRB-A267) approved the protocols for the research. The studies were conducted according to the guidelines of the Declaration of Helsinki and Strengthening The Reporting of Genetic Association Studies (STREGA): An extension of the STrengthening the Reporting of OBservational studies in Epidemiology (STROBE) statement recommendations [21].

### 2.2. Participants

The study involved 2479 individuals (194 chess players, 119 PhD degree holders, and 2166 controls; 1417 males and 1062 females) from Kazakh, Russian and Tatar ethnicities. The Russian subjects were all Caucasians of Eastern European descent, while Kazakhs and Tatars were descendants of Turkic origin living in the Republic of Kazakhstan and Republic of Tatarstan (region of the Russian Federation), respectively. Age and sex characteristics of all cohorts are presented in Table 1. Chess players were classified as non-elite (51 Kazakhs, 42 Russians, 42 Tatars), sub-elite (8 Kazakhs, 16 Russians, 26 Tatars) and elite (9 Russian international chess grandmasters ranked among the 80 best chess players in the world). All PhD (Candidates of Sciences, according to the Russian system) holders earned their degrees in the STEM fields, including medicine (*n* = 10), biology (*n* = 49), chemistry (*n* = 39), technology (*n* = 8), and physics/mathematics (*n* = 13).

### 2.3. Genetic Analysis

#### 2.3.1. Russian and Tatar Samples

DNA samples from Russian and Tatar individuals were obtained from leukocytes (venous blood) or epithelial mouth cells. Four ml of venous blood were collected in tubes containing EDTA (Vacuette EDTA tubes, Greiner Bio-One, Kremsmünster, Austria). DNA samples were transported to the laboratory at 4 °C, and DNA was extracted on the same day. DNA extraction and purification from blood samples were performed using commercial kits (Techno-sorb), according to the manufacturer’s instructions (Technoclon, Moscow, Russia), and included chemical lysis, selective DNA binding on silica spin columns, and ethanol washing. DNK-sorb-A kit (Central Research Institute of Epidemiology, Moscow, Russia) was used for the DNA extraction and purification from epithelial mouth samples, according to the manufacturer’s instructions [22].

Genotyping of the *KIBRA* gene rs17070145 polymorphism was performed using three different methods, including microarray technology [23,24], single-stage PCR followed by hybridization on a hydrogel biochip [25], and a newly designed Restriction Fragment Length Polymorphism method (RFLP). The assay for the microarray analysis required 200 ng of DNA sample as input with a concentration of at least 50 ng/µL. Exact concentrations of DNA in each sample were measured using a Qubit Fluorometer (Invitrogen, Waltham, MA, USA). The genotyping process was performed using HumanOmni1-Quad BeadChips (Illumina, San Diego, CA, USA) to genotype the *KIBRA* gene polymorphism. All further procedures were performed according to the instructions of the Infinium High-Density Assay.

PCR primers for the RFLP analysis were forward 5′-CTAATGCCAGGACGTCATGGCAG-3′ and reverse 5′-ATCTCTTGACCCAGTATAAAAGGA-3′ (Litech, Moscow, Russia), generating a fragment of 214 bp. PCR was conducted on a multicanal amplificator Tercyk (DNA Technology, Moscow, Russia). PCR products were digested with Tru9 I (SibEnzyme, Novosibirsk, Russia) for 12 h at 65 °C, producing 173- and 41-bp fragments for allele T and a 214-bp fragment for allele C. Fragments were then separated by 8% polyacrylamide gel electrophoresis, stained with ethidium bromide, and visualized in UV light. All genotyping analyses were conducted blind to participant identity.

#### 2.3.2. Kazakh Samples

Genomic DNA was extracted from a buccal swab using a QIAamp DNA Mini kit (Cat No. 51306; Qiagen, Germany) according to the manufacturer’s instructions. Briefly, ari-dried cotton buccal swabs post-collection were cut into a 2 mL tube and resuspended into 400 µL PBS followed by lysis with Proteinase K and AL lysis buffer at 56 °C for 10 min. The digested samples were treated with AL buffer and 100% ethanol followed by washes with wash buffer on the QIAamp mini spin column and eluted with 30 µL nuclease-free water. DNA extracted was quantified using NanodropTM (Thermo Fisher Scientific, Waltham, MA, USA). Approximately, 10 ng of DNA was used for the targeted sequencing using Fluidigm Access Array.

The rs17070145 SNP (C/T) was genotyped using Targeted Next Generation Sequencing. First, primers were designed to cover the SNP rs17070145 (chr5: 167845791, GRCh37/hg19) in the *KIBRA* gene. The primer sequences used for genotyping are F: 5′-TACTCCCAGCACACACCTC-3′ and R: 5′-GTTGGCAGATGGAACCCGT-3′. The primers were evaluated using control DNA samples and the expected size was validated using agarose gel electrophoresis. Then, the primers were tagged with Fluidigm-specific tag sequences CS1: ACACTGACGACATGGTTCTACA for the forward’s primer and CS2: TACGGTAGCAGAGACTTGGTCT for the reverse’s primer. PCR was performed with at least 5 ng of DNA from each sample using Fast Start High Fidelity master mix (Roche, Switzerland). The amplified PCR products were further purified using ExoSAP-IT (Invitrogen, USA) and amplified with the tagged target-specific primers on the 48.48 Access Array integrated Fluidic circuit (IFC) using a Fast start high fidelity master mix (Roche). The prepared amplicons were allied with 48.48 Access Array IFC barcodes, purified with AMPure XP beads (Beckman Coulter, Brea, CA, USA) and quantified using High sensitivity DNA assay kit on Bioanalyzer (Agilent, Santa Clara, CA, USA) following the manufacturer’s instructions. The libraries were further proceeded using emulsion PCR with Ion Sphere^TM^ particles with Ion Template OT2 kit in Ion OneTouch™ ES system following the manufacturer’s instructions (Thermo Fisher Scientific, Waltham, MA, USA). All samples were pooled and sequenced using the Ion 520™ Chip on the Ion S5 XL Semiconductor sequencer following the manufacturer’s instructions (Thermo Fischer).

The resulting reads were processed and quality control was assessed using the Ion Torrent Software Suite version 5.12.3. The burrows wheeler aligner (BWA) was used to align the raw sequencing reads to the reference genome GRCh37/hg19. SNP calling and genotyping of the rs17070145 SNP was performed using an in-house bioinformatics pipeline that incorporates SAMtools mpileup commands. Samples showing low coverage depth were excluded from the downstream analysis.

### 2.4. Statistical Analyses

Statistical analyses were conducted using GraphPad InStat (GraphPad Software, Inc., San Diego, CA, USA) software. Genotype frequencies of cases (chess players and PhD holders) and controls were tested for compatibility with Hardy–Weinberg equilibrium (HWE). Genotype distribution and allelic frequencies between cases and controls were compared using χ^2^ or Fisher’s exact tests. To perform the meta-analyses the Cochrane Review Manager (RevMan) (London, UK) version 5.3 was used. Random effects models were applied. Odds ratios (OR) with 95% confidence intervals (CI) were estimated using the Mantel-Haenszel method. The heterogeneity degree between the studies was assessed with the I^2^ statistic. *p* values < 0.05 were considered statistically significant. Where appropriate, Bonferroni’s correction for multiple testing was performed by dividing the *p* value (0.05) by the number of tests.

## 3. Results

### 3.1. Association of the KIBRA Gene Variant with Chess Player Status

**Figure 1:**
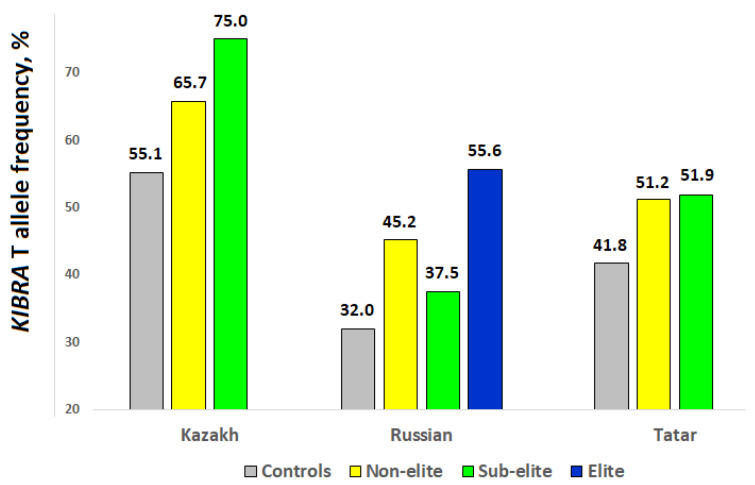
*KIBRA* gene rs17070145 T allele frequency in controls and chess players stratified by the level of achievement.

In Kazakh, Russian, and Tatar groups of chess players and controls, the *KIBRA* gene rs17070145 polymorphism met Hardy-Weinberg expectations (*p* > 0.05 in all groups tested separately). Frequencies of the rs17070145 T allele were significantly higher in Kazakh (66.9 vs. 55.1%; OR = 1.65, 95% CI 1.07–2.56; *p* = 0.024, χ^2^ = 4.64), Russian (44.8 vs. 32.0%; OR = 1.73, 95% CI 1.22–2.45; *p* = 0.0027, χ^2^ = 9.00), and Tatar (51.5 vs. 41.8%; OR = 1.48, 95% CI 1.03–2.11; *p* = 0.035, χ^2^ = 4.22) chess players compared with ethnically matched controls (Table 2). However, only in Russian chess players did the results remain statistically significant after correction for multiple testing. Across all groups, sub-elite/elite chess players tended to have higher frequencies of the T allele compared with non-elite chess players (Figure 1). In addition, none of the Russian international chess grandmasters (0 vs. 46.3%; OR = 16.4; *p* = 0.005, χ^2^ = 6.0) were carriers of the CC genotype (which was the most prevalent genotype in the Russian controls).

**Figure 2:**
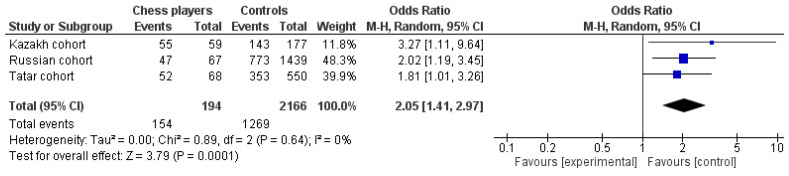
Meta-analysis for association studies for the *KIBRA* gene and chess player status. The prevalence of the rs17070145 CT/TT genotypes has been shown in chess players. The purple squares represent the ratio of CT/TT genotypes in chess players for each individual study. The black diamond represents the pooled ratio of CT/TT genotypes in all chess players and its 95% CI.

Given that allelic frequencies between three populations were significantly different (*p* < 0.0001), we performed a meta-analysis. The pooled OR favoring chess players compared with controls was 2.05 (95% CI 1.41–2.97, *p* = 0.0001 for the random effect model of meta-analysis) for the carriage of the T allele (i.e., rs17070145 CT/TT genotypes). No heterogeneity between studies (I^2^ = 0%; *p* = 0.64) was observed (Figure 2). These results indicate that carriage of the *KIBRA* CT/TT genotypes is strongly associated with chess player status.

### 3.2. Association of the KIBRA Gene Variant with PhD Status

**Figure 3:**
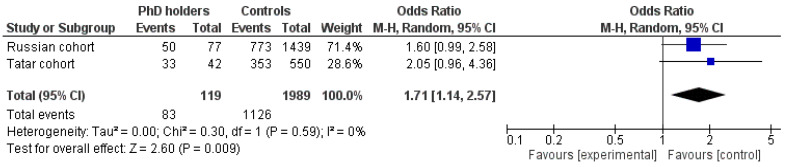
Meta-analysis for association studies for the *KIBRA* gene and PhD status. The prevalence of the rs17070145 CT/TT genotypes has been shown in PhD holders. The purple squares represent the ratio of CT/TT genotypes in PhD holders for each individual study. The black diamond represents the pooled ratio of CT/TT genotypes in all PhD holders and its 95% CI.

The *KIBRA* gene rs17070145 polymorphism met Hardy-Weinberg expectations in Russian and Tatar PhD holders (*p* > 0.05). The frequency of the rs17070145 T allele was significantly higher in Russian PhD holders (40.9 vs. 32.0%; OR = 1.47, 95% CI 1.06–2.05; *p* = 0.026, χ^2^ = 4.94) compared with Russian controls, but not in Tatar PhD holders (48.8 vs. 41.8%; OR = 1.33, 95% CI 0.85–2.07; *p* = 0.252, χ^2^ = 1.29) (Table 2). The pooled OR favoring PhD holders compared with controls was 1.71 (95% CI 1.14–2.57, *p* = 0.009 for the random effect model of meta-analysis) for carriage of the T allele. No heterogeneity between studies (I^2^ = 0%; *p* = 0.59) was observed (Figure 3).

## 4. Discussion

The aim of the present study was to compare allelic frequencies of the *KIBRA* rs17070145 C/T polymorphism between chess players, PhD holders, and controls. As hypothesized, the favorable T allele was associated with chess player status and PhD status in science, and this finding was replicated across three cohorts. The latter replications are essential to confirm associations in genetic studies, when there are a limited number of individuals, such as in the case of elite athletes [26,27,28]. To our knowledge, then, this is the first study demonstrating that ability in chess and science may depend on a specific gene variant—the *KIBRA* rs17070145 SNP, previously associated with working memory, spatial ability, and other cognitive traits.

Although the rs17070145 C/T polymorphism is intronic, it may be functional. Indeed, according to the GTEx portal [29], this SNP is a splicing quantitative trait locus (sQTL), with the T allele associated with significantly (*p* = 1.3 × 10^−10^) altered changes in the splicing ratios of transcripts in brain tissue. Furthermore, Piras et al. [30] reported that the TT genotype was associated with overexpression of genes involved in the MAPK signaling pathway (*MAPK8IP1*, *GADD45B*, *DUSP2*, *DUSP5*, *PRKCG*, *NR4A1*, *MAPK11*, *HSPA1A*, *HSPA1B* and *HSPB1*) in the human hippocampus of 22 neuropathologically normal individuals. Interestingly, the MAPK signaling pathway has been previously linked with memory and learning processes [31,32].

Studies indicate that the *KIBRA* rs17070145 SNP is associated with brain metabolism and structure. Accordingly, Palombo et al. [33] demonstrated that T allele carriers had a larger hippocampal volume (cornu ammonis and dentate gyrus subregions) relative to noncarriers (i.e., CC genotype) among young people. This observation was confirmed in another study of older adults [34]. In addition, the *KIBRA* T allele has been linked with greater gray matter volume in the prefrontal cortex and parahippocampal cortex [35]. Furthermore, T allele carriers have been shown to exhibit higher glucose metabolism than carriers of the CC genotype in posterior cingulate and precuneus brain regions [36]. Lower cerebral glucose metabolism is considered one of pre-symptomatic endophenotypes associated with dementia [36,37]. Indeed, the T allele has been reported to protect against cognitive decline with age [38,39] and Alzheimer’s disease [36,40].

Chess is a board game that falls under the family of mind sports and requires a high level of spatial visual processing, attention, and working memory [41]. The heritability of skill in chess has been estimated at 48% [42]. Consequently, our findings that the *KIBRA* T allele and CT/TT genotypes are over-represented in three independent cohorts of chess players (and notably in all 9 international chess grandmasters) are supported by the fact that the T allele is associated with better attention/concentration [43], working memory [4] and spatial-related traits [8,9,10]. It should be noted that psychogenetic research in sport until now has been limited to studies involving power, endurance, combat, and game sports athletes only, with a focus on stress resilience (*COMT* rs4680), reaction time (*APOE* ε2/ε3/ε4, *KIF27* rs10125715, *APC* rs518013, *TMEM229A* rs7783359, *LRRN3* rs80054135), memory (*APOE* ε3/ε4, *BDNF* rs6265), sensation seeking (*DRD3* rs167771, *DRD4* rs1800955), anxiety (*ACE* I/D, *COMT* rs4680, *MAOA* VNTR polymorphism), aggressiveness (*MAOA* VNTR polymorphism), and motivation (*MAOA* VNTR polymorphism) [44,45,46]. Therefore, the present study, the first genetic research into mind sports athletes, represents an important step forward in the field.

In a study of the personality traits of 2015 scientists and 78,753 nonscientists, it was demonstrated that scientists scored higher than nonscientists on tough mindedness (a disposition to be analytical—drawing conclusions, and making decisions, based on logic, facts, and data) and openness (to innovation, novel experiences, and new learning) [47]. Scientists also need to be creative and have spatial ability [12,48]. Analytical ability and openness to experience positively correlate with intelligence and creativity [49,50]. There is also a positive relationship between working memory and other cognitive traits, such as creativity, analytic problem-solving, fluid intelligence, and spatial ability [19,20,51,52]. Given the above, it is, therefore, unsurprising that the PhD holders in our study had a greater frequency of the favorable *KIBRA* T allele and CT/TT genotypes compared with the general population. Indeed, in line with our findings that possessing the CT/TT genotype was associated with a greater disposition to earn a PhD in Russian and Tatar participants, bioinformatic analysis revealed that the *KIBRA* T allele was positively associated (*p* = 0.00089) with educational attainment in the UK Biobank cohort (*n* = 240,547) [53]. Importantly, there are at least 3952 known genome-wide significant SNPs associated with educational attainment [54], which may be considered in future research as potential candidate genetic markers related to achievement in science.

Against the backdrop of this study’s novel findings, there are some limitations that should be acknowledged. First, the size of the sample of PhD holders and chess players was moderate. Thus, although our results were further supported through meta-analyses, extension to and replication within groups of differing geographic ancestry is needed to translate these findings more broadly. Second, although our hypothesis—that the T allele of the *KIBRA* rs17070145 SNP would be associated with chess player status and PhD status in science—was based on evidence for the importance of spatial ability and working memory, we did not actually assess the latter traits in this study. Given the preceding lines, a third limitation is then that our comparison was between chess players and PhD holders with presumably high levels of key cognitive traits and controls, whose cognitive traits were essentially unknown. A fruitful (and potentially more powerful) future design would thus be to compare allelic frequencies of the *KIBRA* gene between individuals with known (measured) *high* and *low* cognitive abilities.

## 5. Conclusions

In conclusion, our results suggest that the rs17070145 C/T polymorphism of the *KIBRA* gene is associated with ability in chess and science, with the T allele exerting a beneficial effect. Nevertheless, given the substantial heritable component of cognitive and personality traits, and that each DNA locus may explain only a very small proportion of phenotypic variance (e.g., ~0.01% to ~1%), there are clearly more—and probably many more—genetic variants associated with ability in chess and science that remain to be identified. We thus caution that more evidence is needed before application of findings such as ours (e.g., via genetic testing) in talent identification in chess and science.

## Figures and Tables

**Table 1:** Characteristics of studied groups.

Groups	Males		Females	
	*n*	Age, Years	*n*	Age, Years
Kazakh chess players	46	17.0 (3.9)	13	16.3 (4.4)
Kazakh controls	111	27.5 (4.6)	66	25.0 (3.6)
Russian chess players	44	22.2 (3.2)	23	22.8 (3.1)
Russian PhD holders	34	27.6 (2.8)	43	30.4 (4.9)
Russian controls	831	27.1 (4.3)	608	27.2 (4.4)
Tatar chess players	48	21.9 (3.8)	20	21.4 (3.2)
Tatar PhD holders	25	30.2 (4.7)	17	32.2 (6.5)
Tatar controls	278	25.7 (4.8)	272	25.1 (4.3)

Data are Mean (SD).

**Table 2:** *KIBRA* rs17070145 genotype distribution and T allele frequency in different cohorts.

Cohort	*n*	*KIBRA* Genotypes			T Allele, %	*p*
		CC	CT	TT		
Kazakh chess players	59	4	31	24	66.9	0.024 *
Kazakh controls	177	34	91	52	55.1	-
Russian chess players	67	20	34	13	44.8	0.0027 *
Russian PhD holders	77	27	37	13	40.9	0.026 *
Russian controls	1439	666	626	147	32.0	-
Tatar chess players	68	16	34	18	51.5	0.035 *
Tatar PhD holders	42	9	25	8	48.8	0.252
Tatar controls	550	197	246	107	41.8	-

* *p* < 0.05, statistically significant differences in allelic frequencies between cases (chess players or PhD holders) and controls.

## Data Availability

The data presented in this study are available on request from the corresponding author.
